# The Role of Extracellular Vesicles in Metabolic Reprogramming of the Tumor Microenvironment

**DOI:** 10.3390/cells11091433

**Published:** 2022-04-23

**Authors:** Eran S. Fridman, Lana Ginini, Ziv Gil

**Affiliations:** 1 Rappaport Family Institute for Research in the Medical Sciences, Technion—Israel Institute of Technology, Haifa 31096, Israel; eran.frid@gmail.com (E.S.F.); lana.ginene@gmail.com (L.G.); 2 Head and Neck Institute, The Holy Family Hospital Nazareth, Nazareth 1641100, Israel

**Keywords:** extracellular vesicles (EVs), exosomes, cancer metabolism, tumor microenvironment (TME), glycolysis

## Abstract

The tumor microenvironment (TME) includes a network of cancerous and non-cancerous cells, together with associated blood vessels, the extracellular matrix, and signaling molecules. The TME contributes to cancer progression during various phases of tumorigenesis, and interactions that take place within the TME have become targets of focus in cancer therapy development. Extracellular vesicles (EVs) are known to be conveyors of genetic material, proteins, and lipids within the TME. One of the hallmarks of cancer is its ability to reprogram metabolism to sustain cell growth and proliferation in a stringent environment. In this review, we provide an overview of TME EV involvement in the metabolic reprogramming of cancer and stromal cells, which favors cancer progression by enhancing angiogenesis, proliferation, metastasis, treatment resistance, and immunoevasion. Targeting the communication mechanisms and systems utilized by TME-EVs is opening a new frontier in cancer therapy.

## 1. Introduction

In 1930, Otto Warburg first described a metabolic phenomenon in cancer cells, which has been known ever since as the Warburg effect [1]. He described the paradoxical finding that the behavior of cancer cells tends towards less efficient glycolysis over mitochondrial oxidative phosphorylation, which seems counterintuitive since the energy yield is lower in the former versus the latter. As a consequence, cancer cells must adapt and fuel themselves with increasing amounts of glucose molecules to meet the demand for adenosine triphosphate (ATP), which is necessary for cellular metabolism and proliferation. This well-recognized fact has led to the use of positron emission tomography (PET) with a radiolabeled analog of glucose (18F-fluorodeoxyglucose, FDG) as a reporter, to visualize and track tumor growth and progression. Warburg initially presumed that this behavior was due to mitochondrial defects that lead to impaired aerobic respiration and consequent dependence on glycolytic metabolism. However, the finding that mitochondrial function is normal in most cancers advocates for a different explanation [2].

It has been understood for the past two decades that the reprogramming of cell metabolism is a central phenomenon in cancer, to the point that it is now considered to be one of the hallmarks of the disease [3]. In this regard, many alternations in central metabolic pathways have been described, including amino acids, nucleotide biosynthesis, fatty acid metabolism, and glucose metabolism. For the latter, several alteration pathways have been identified and their behaviors studied, including the hexosamine synthesis pathway (HSP), the pentose phosphate pathway (PPP), and the serine biosynthesis pathway (SBP) [4]. The PPP, for example, branches off glycolysis after glucose has been phosphorylated. The products of PPP are ribulose-5-phosphate (R5P), a precursor of nucleotide de novo synthesis, and NADPH, which is necessary for fatty acid synthesis and aids in maintaining redox homeostasis and protecting cancer cells from oxidative stress [5]. Induced PPP is observed in many cancer types, including colorectal, breast, and lung cancers, as well as hepatocellular carcinoma [6,7].

Another well-described change is that which occurs in regard to glutamine metabolism. Glutamine is a nitrogen donor for amino acid and nucleotide synthesis. It can also be used to produce TCA cycle intermediates. Glutamine also contributes to fatty acid synthesis, which is necessary for biosynthesis to occur in proliferating cancer cells. This pathway is particularly active during aerobic glycolysis (Warburg effect) when cancer cells transform most pyruvate into lactate, rather than acetyl-CoA. Under these circumstances, most of the acetyl-CoA in cancer cells is acquired from the glutamine-TCA cycle axis [8,9].

Several mechanisms that have already been described as contributing to the alteration of metabolic processes and thus the growth of cancer have focused on oncogenes. Perhaps the most well-known oncogene is RAS, which has been found to be altered in many cancer types. A variety of studies have revealed that mutant RAS controls the metabolisms of glucose [10], glutamine [11], and lipids. Similarly, other oncogene-driven metabolic alternations have been described for MYC, TP53, HIF-1, EGFR, and BRAF [12,13,14]. Other explanations for altered metabolic states in cancer have been ascribed to the effects of the tumor microenvironment (TME). As a tumor grows, it exceeds the diffusion limits of its local blood supply, leading to cellular hypoxia and stabilization of the hypoxia-inducible transcription factor (HIF) [15]. Under transcriptional supervision of HIF, cellular metabolism shifts toward glycolysis via increased expression of glycolytic enzymes, glucose transporters, and mitochondrial metabolism inhibitors.

### 1.1. Tumor Microenvironment and Metabolic Adjustment

The TME includes a network of cells and structures that surround tumor cells. Included here are tumor cells, neighboring nonmalignant cells (e.g., immune cells, cancer-associated fibroblasts, endothelial cells, adipocytes, and mesenchymal stem cells), the extracellular matrix, the nearby vasculature, and signaling molecules (cytokines, growth factors, hormones, etc.). On their own, cancer cells cannot establish and maintain the disease alone. Rather, resident and recruited noncancerous cells operate as accomplices in tumor progression [16].

TME cells are affected by cancer cells and undergo metabolic modifications as a result. For instance, the process of tumor aerobic glycolysis releases increased amounts of lactate into the extracellular space, which decreases the immunoresponsiveness of dendritic [17] and T cells [18] and depresses monocyte migration [19]. In turn, lactate stimulates macrophage polarization towards tumor-associated macrophages (TAMs), which contribute to tumor progression [19]. TME cells have also been shown to fuel cancer cells. An example of this is known as the “reverse Warburg effect” in which TME cells are metabolically altered toward a lactate-producing glycolytic phenotype that is expelled from the cell into the TME. The associated cancer cells are reprogrammed toward oxidative phosphorylation (OXPHOS) and the extracellular lactate is taken up by tumor cells and used to replenish the TCA cycle [20,21,22]. TME cells can also transfer amino acids, such as glutamine [23] and alanine [24], to replenish cancer cells.

Recently, evidence has accumulated on the effects of extracellular vesicles on metabolic pathways in cancer that favor disease progression.

### 1.2. Extracellular Vesicles

Extracellular vesicles (EVs) are lipid bilayer membrane particles surrounding a cytosol compartment. EVs can form through outward budding of the plasma membrane or via an intracellular endocytic trafficking pathway, which involves the fusion of multivesicular late endocytic compartments with the plasma membrane. Accordingly, EVs can be classified according to their biogenesis and biophysical/biochemical characteristics. Subtypes include intracellular formed exosomes (50–200 nm), which are secreted after the fusion of multivesicular bodies with the cell surface; microvesicles (100–1000 nm), which are formed through outward budding of the plasma membrane, whose shed midbody remnants are released during cytokinesis (200–600 nm); and apoptotic bodies (100–5000 nm), which are released during apoptosis [25].

Due to the difficulty in assigning an EV to a particular biogenesis pathway, the International Society for Extracellular Vesicles (ISEV) recommends the use of “EV” as a catchall term for these types of vesicles [26]. The organization recommends classifying EVs according to their physical attributes (size and density), different biochemical compositions, and surface charges. Nevertheless, there is great diversity in the literature regarding the EV nomenclature that has arisen as a result of the several separation and isolation methods that have been employed, as well as the lack of a thorough characterization of EVs. As it is beyond the scope of this review to detail the still-evolving landscape of EV isolation and characterization methodologies, let alone the many terms that are used to describe the varied spectrum of vesicles that have been studied, to avoid inaccuracies and facilitate comprehension, in this article we will simplify the matter and refer to all of these as EVs.

During biogenesis, an EV can selectively capture cell-specific proteins, lipids, DNA, and RNA species (mRNA, miRNA, tRNA, lnRNA, etc.), which may become a part of the “molecular signature” of the EV membrane or its cargo [27,28]. However, the mechanism involved that effectuates such selective packaging remains unknown [29]. The rising interest in EVs is related to their ability to induce phenotypic changes in recipient cells. In particular, EVs can transmit information to recipient cells by acting on the cell surface without delivering the cargo they contain. For example, B cell lymphocyte-released EVs induce antigen-presenting responses in T cells via MHC class-II without fusing to the plasma membrane [30]. However, the principal modus operandi of EVs is to deliver cargo upon their internalization into recipient cells [31]. 

EVs are capable of modifying whole-body metabolism. For example, macrophage-derived EVs in adipose tissue have been shown to alter whole-body insulin sensitivity [32]. EVs derived from endothelial cells transferred proteins and lipids to adipocytes. Moreover, this transport was modulated by systemic energy state (fasting and obesity) [33]. EVs play a a fundamental role in many steps leading to tumor progression. These include effects on cell proliferation [34], angiogenesis [35], thrombotic events [36], immunoescape [37], metastasis [38], and therapeutic resistance [39,40].

The remainder of this review will focus on EVs in the TME and their role in shaping the metabolic landscape of cancer and stromal cells.

## 2. Metabolic Reprogramming by Cancer-Derived Extracellular Vesicles

Extracellular vesicles have been shown to transport proteins, lipids, and nucleic acids. Glycolytic enzymes are commonly found in proteomic profiling of EVs from different origins. Most of these enzymes are listed among the top 100 proteins that have been identified in EVs [41]. Not only do EVs convey cargo that reprograms metabolic pathways, but they are also metabolically active themselves. They function as independent, extracellular metabolic units, which can modify the concentrations of critical nutrients, with the potential to affect the physiology of their microenvironment. For example, prostasomes, a family of EVs secreted by the prostate, can produce extracellular adenosine triphosphate (ATP) [42], while neural stem/progenitor cell (NSC) EVs exhibit L-asparaginase enzymatic activities that affect the consumption/release of metabolites in the extracellular compartment [43].

**Figure 1:**
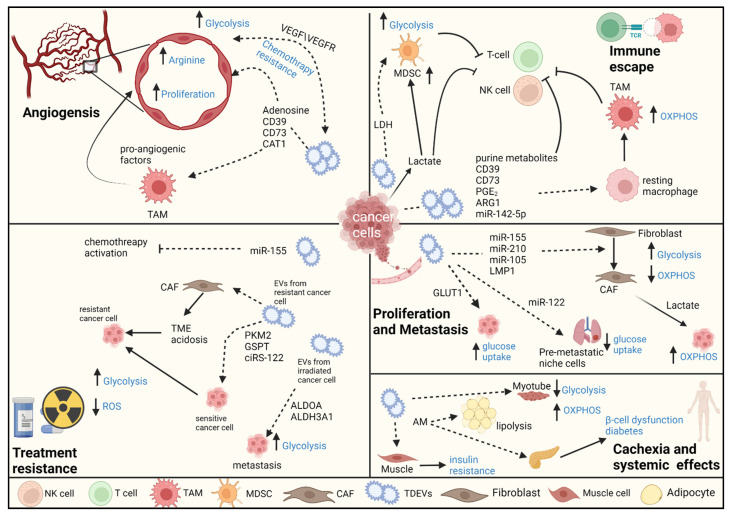
Tumor microenvironment metabolic reprogramming by tumor-derived extracellular vesicles. Tumor-derived EVs (TDEVs) have been implicated in almost all tumor aspects, including cell proliferation and metastasis, angiogenesis, immunoescape, and therapy resistance. TDEVs transfer cargo into endothelial cells that increase arginine metabolism and glycolysis, which promotes endothelial proliferation and angiogenesis. TDEVs transfer glycolytic enzymes and genetic material from treatment-resistant cancer cells to sensitive cancer cells, which thereby enhances glycolysis, causes a decrease in ROS, and increases chemotherapy metabolism, thus conferring treatment resistance. EVs carry and deliver purine metabolites, genetic material, and glycolytic enzymes that increase glycolysis in MDSC and polarize macrophages to TAMs, which, together with TDEVs, have a direct effect on inhibiting the immunoresponse to cancer. TDEVs activate fibroblasts to CAFs, which induces glycolysis in CAFs and, in turn, promotes proliferation and metastasis. TDEVs act on cancer cells to increase glucose uptake while suppressing glucose uptake by premetastatic niche cells, which promotes metastasis. TDEVs travel beyond the immediate TME to inhibit glycolysis and enhance OXPHOS in myoblasts, which disrupts myotube differentiation and partly explains cachexia. TDEVs induce insulin resistance in skeletal muscle cells and transfer AM to induce lipolysis and β-cell dysfunction, which is manifested as cancer-related diabetes. NK cell—natural killer cell; TAM—tumor-associated macrophage; MDSC—myeloid-derived suppressor cell; CAF—cancer-associated fibroblast; TME—tumor microenvironment; AM—adrenomedullin; ROS—reactive oxygen species; OXPHOS—oxidative phosphorylation. Dashed arrow—transferred EV cargo.

Indeed, different EVs carry unique substances that can adjust the metabolic landscape of recipient cells in specific ways, and thereby support tumor progression and confer systemic effects (e.g., cachexia). In Figure 1, we present an overview of how tumor-derived EVs (TDEVs) support cancer progression.

### 2.1. Proliferation and Metastasis

#### 2.1.1. Effect of TDEVs on Stromal Cells

Exposure of human adult dermal fibroblasts to human-melanoma-derived EVs leads to an increase in aerobic glycolysis, a decrease in OXPHOS in dermal fibroblasts, and a consequent increase in extracellular acidification [44]. The acidic environment encourages the release of EVs in metastatic melanoma cell lines and changes the content of those selfsame EVs [45]. EV protein analysis has revealed enrichment for different pathways responsible for melanoma migration and invasion, as well as metastasis and survival. miR-155 and miR-210 were found to be responsible for observed changes in glycolysis and OXPHOS; changes that were reversed through the use of anti-miR to decrease the expression of these microRNAs (miRNAs) [44]. Additionally, absent any other process, acidification of the extracellular environment has been found to promote the metastasis of melanoma cells [46]. Breast cancer-secreted, EV-encapsulated miR-105 activates signaling in cancer-associated fibroblasts (CAFs) to induce metabolic reprogramming. Furthermore, activated CAFs display different metabolic features in response to changes in the metabolic environment. In a nutrient-sufficient environment, miR-105-reprogrammed CAFs enhance glucose and glutamine metabolism to fuel adjacent cancer cells. In a nutrient-deprived environment, CAFs detoxify metabolic wastes, including lactic acid and ammonium, by converting them into energy-rich metabolites. Thus, the miR-105-mediated metabolic reprogramming of stromal cells contributes to sustained tumor growth by acclimatizing the metabolic TME, thus resulting in increased proliferation [47].

In nasopharyngeal carcinoma (NPC), secreted EVs are packed with latent membrane protein 1 (LMP1)-activated normal fibroblasts to become cancer-associated fibroblasts (CAFs). In CAFs, the delivered LMP1 activated the NF-κB/p65 pathway, which in turn increased aerobic glycolysis and autophagy in CAFs. At the same time, glucose and lactate levels decreased in NPC cancer cells, thus supporting the notion of the “reverse Warburg effect.” Finally, EV-packaged, LMP1-activated CAFs have been found to promote tumor cell proliferation, migration, and radiation resistance. In vivo, EV-packaged, LMP1-activated CAFs increased tumor volume and increased the levels of premetastatic niche factors (fibronectin, S100A8, and VEGFR1) in lung and liver tissue [48]. Similarly, oral cavity squamous cell carcinoma (OCSCC) shed EVs that stimulate the transformation of the normal human gingival fibroblast phenotype into CAFs. In turn, CAFs undergo degradation of caveolin-1 (CAV1) through the ERKl/2 activation pathway. CAV1 degradation further induces the metabolic switching to aerobic glycolysis in fibroblasts. CAFs absorb more glucose and produce more lactate, and coculture CAFs with OCSCC-promoted cancer cell migration and invasion [49].

#### 2.1.2. Effects of TDEVs on Cancer Cells

Kaposi’s sarcoma-associated herpesvirus (KSHV) is the etiological agent of Kaposi’s sarcoma (KS). KSHV-infected cells specifically transfer virus-encoded mRNAs to surrounding cells via EVs, which stabilizes hypoxia-induced factor 1 alpha (HIF1α) in noninfected cells. HIF1α is a known regulator of glucose metabolism and its stabilization results in a metabolic shift toward aerobic glycolysis in surrounding noninfected cells. This transforms the noninfected EV recipient cells into “feeder cells,” secreting energy-rich metabolites, such as lactate and pyruvate, and supporting the growth of infected (EV-shedding) cells [50]. In a different study, EVs derived from KRAS-mutated colorectal cancer cells (CRC) conferred a Warburg-like effect on colonic epithelial cells in vitro and in vivo, which resulted in cell proliferation. These EVs carry GLUT1 (glucose transporter), which contributes to metabolic changes in recipient cells [51].

Retinoblastoma (RB) is the most common intraocular malignancy in childhood. When EVs released from metastatic RB cell lines (vitreous seeding) have been compared to EVs from nonmetastatic RB cell lines, the former have been observed to carry several upregulated proteins involved in glycolysis, glucose catabolism, and amino acid synthesis [52].

TDEVs can reprogram both cancer and neighboring stromal cell metabolism, as well as systemic energy metabolism. For example, breast-cancer-secreted EVs carry high amounts of miR-122, which suppresses glucose uptake by premetastatic niche cells in vitro and in vivo by downregulating pyruvate kinase, a glycolytic enzyme [53]. This facilitates tumor metastasis, whereas the inhibition of miR-122 reduces metastasis in vivo.

### 2.2. Immunoescape

The ability of a tumor to suppress immunoresponsiveness is one of the hallmarks of cancer [3]. Several mechanisms have been described to account for the ability of EVs to promote immunosuppression. TDEVs can directly induce apoptosis of immune cells [54] and trigger the differentiation of myeloid cells to myeloid-derived suppressor cells (MDSCs) [55], as well as inhibiting the cytolytic activity of NK cells [56] and the exosomal expression of programmed death ligand-1 (PDL1) [57].

#### 2.2.1. Suppression of Natural-Killer (NK) Cells

As described in the previous section, EVs stimulate lactate production and secretion into the extracellular space. This causes extracellular acidosis, which inhibits NK proliferation and function [58], and the impairment of cytolytic activity and cytokine secretion in tumor-specific CD8+ T lymphocytes [59]. Lactate itself can block the proliferation, tumor infiltration, and cytokine production of T cells, and inhibit the cytotoxic activity of NK-, NKT-, and CD8+ cells, as well as increase the number of MDSCs that inhibit NK cytotoxicity [60].

#### 2.2.2. Suppression of T Cells

Another mechanism of T cell immunosuppression involves extracellular adenosine binding to A1/A2A/A2B/A3 adenosine receptors on the surface of most immune cells [61]. TDEVs can influence adenosine-induced immunosuppression in several different ways. For example, CD39/CD73 are phosphatases that catalyze the conversion of ATP to AMP and the hydrolysis of AMP to adenosine. These phosphates are expressed by EVs from a variety of cell lines, including the bladder, colorectal, prostate, and mesothelioma cells lines [62]. This EV enzymatic activity generates extracellular adenosine through the degradation of extracellular ATP, which consequently inhibits T cell activation. Similarly, EVs derived from head and neck squamous cell carcinoma (HNSCC) have been shown to trigger extracellular inosine production (adenosine metabolite) via regulatory T cells (Tregs) [63]. EVs have also been shown to contain adenosine, which acts directly on T cell membrane adenosine receptors, thereby suppressing T cell activity [64]. Moreover, EVs from HNSCC patients were shown to carry purine metabolites, such as adenosine, inosine, and xanthine, thereby expanding the immunosuppressive potential of EVs [65]. In a separately described mechanism, prostate cancer-derived EVs were found to carry prostaglandin E2, which triggers CD73 expression in dendritic cells, thus resulting in T cell inhibition in an adenosine-dependent manner [66].

Ovarian cancer EVs have been shown to carry arginase 1 (ARG1), an enzyme that catalyzes the degradation of semi-essential L-arginine to L-ornithine and urea. ARG1 impairs T cell functions [67], whereas the depletion of arginine arrests T cell cycle progression and inhibits IFN-γ production [68]. Czystowska-Kuzmicz et al. have shown in vivo that ARG1-containing EVs from ovarian carcinoma suppress peripheral T cell proliferation. Furthermore, increased ARG1 expression in mouse ovarian cancer cells was revealed to have an association with accelerated tumor progression that can be blocked through the use of an arginase inhibitor [67]. Immunosuppression is also mediated by the transfer of miRNAs from cancer cells to neighboring cells. Cervical squamous cell carcinoma (CSCC) secreted EVs containing miR-142-5p and, upon uptake by lymphatic endothelial cells (LECs), induced the expression of indoleamine 2,3-dioxygenase (IDO), which is an enzyme that converts the essential amino acid, tryptophan, into kynurenine along what is known as the Kyn pathway. Elevated expression of IDO suppresses and exhausts CD8+ T cells [69].

#### 2.2.3. Macrophage Polarization

A hypoxic tumor environment stimulates the secretion of EVs and changes the protein content of hypoxic TDEVs compared to normoxic TDEVs [70]. These hypoxic EVs promote M2-like polarization of tumor-infiltrating monocytes/macrophages and enhance mitochondrial OXPHOS. The M2 macrophages indicate a higher expression of COX-2, PGES-1, and IL-6, which have established roles in host immunosuppression and tumor growth [71].

In contrast to the aforementioned enhanced OXPHOS in M2 macrophages, EVs derived from pancreatic cancer (PC) cells with SMAD4 deletion (a mutation that exists in 55% of tumors and carries a worse prognosis), create an immunosuppressive myeloid cell background by increasing calcium fluxes and glycolysis through the transfer of SMAD4-related, differentially expressed miRNAs and proteins [72]. Specifically, EVs transfer the glycolytic enzyme activity of lactate dehydrogenase (LDH) [72]. This demonstrates the complexity and sometimes contradictory effects of EVs on metabolic phenotypes in different cancer models.

Immunotherapy is a new player in cancer treatment, involving drugs that activate and use the immune system to attack cancer cells [73]. One drug that has shown favorable results targets the immune checkpoint programmed cell death protein 1 (PD-1), which is a co-receptor expressed on an activated T cell. PD1, upon activation by its ligand PD-L1, transmits a negative costimulatory signal in T cells, resulting in the inhibition of T cell proliferation, cytokine production and release, and cytotoxicity [74]. Anti-PD-1 antibodies block the PD-1 signaling pathway, preventing the PD-1-mediated attenuation of T cell receptor signaling, which promotes the rejuvenation of exhausted PD-1+CD8+T cells, resulting in improved immunoresponsiveness [75]. The precise antitumor immunity mechanism that is involved in PD-1 blockade is not fully understood [76]. Other than restoring T cell activity through T cell receptor signal modulation, PD-1 signaling blockade also reverses the associated metabolic reprogramming, which in part mediates the reappearance of tumor antigen-specific T cells. Treatment with anti-PD-1 monoclonal antibodies induces metabolic changes, including those involving oxidative phosphorylation, glycolysis, respiratory electron transport, TCA cycle, and pentose phosphate pathways [77].

EVs can carry PD-L1 on their surface or as cargo within the vesicle. For example, metastatic melanoma releases high levels of EVs that carry PD-L1 on their surface [78]. PD-L1-carrying EVs have been found to inhibit the proliferation, cytokine production, and cytotoxicity of CD8 T cells. Moreover, neutralizing PD-L1-carrying EVs by pretreating them with anti-PD-L1 antibodies or by genetically editing PD-L1 expression [79] substantially diminished these effects. Similarly, EVs isolated from the plasma of patients with head and neck SCC have been observed to carry PD-L1, with a correlation being established between PD-L1 levels and disease severity. Blocking PD-L1 carried by EVs attenuated immunosuppression [80]. The negative effect of EV PD-L1 on immunoresponsiveness to tumors has also been demonstrated in melanoma [52], Wilms’ tumor [81], gastric cancer [82], lung cancer [83], and glioblastoma [84], among others.

A recent study linked EV PD-L1 to metabolic reprogramming in the TME. Morrissey et al. have shown that lung adenocarcinoma-derived EVs polarize macrophages in the premetastatic niche toward an immunosuppressive state. TDEVs increase PD-L1 expression in macrophages through metabolic reprogramming towards the glycolytic phenotype, which is accompanied by increased glucose uptake, thus establishing a premetastatic, immune-privileged TME [85]. Additional research is needed to further elucidate the activities and interactions of EVs, and the immunotherapeutic roles they may be able to play in the metabolic reprogramming of the TME.

### 2.3. Treatment Resistance

Treatment resistance is a major problem in cancer therapy and research. Chemotherapeutic resistance can be broadly divided into several categories—drug transport and metabolism, alternations in drug targets, and adaptive response (DNA repair) [86]. Patel et al. have shown that EVs secreted by gemcitabine-treated PC cells promote chemoresistance. This was achieved via upregulation of the detoxifying enzymes of reactive oxygen species (ROS) and the decreased expression of miR-155-mediated deoxycytidine kinase (DCK), which is an enzyme that phosphorylates gemcitabine into its active metabolite. Accordingly, EVs decreased the conversion of gemcitabine to its active metabolite.

Altered metabolism that gives rise to treatment resistance via EVs has also been described for other tumors. Adriamycin-resistant breast cancer cells and their corresponding exosomes display a higher expression of glutathione S-transferase P1 (GSTP1), which belongs to the family of phase II metabolic enzymes responsible for the detoxification of several anticancer drugs by conjugating them with glutathione. GSTP1-containing EVs conferred drug resistance from resistant cancer cells to sensitive cancer cells [87]. In CRC, exosomes from oxaliplatin-resistant cells delivered circular RNA (circRNA)-122 to sensitive cells, thereby promoting glycolysis and drug resistance through miR-122 sponging and pyruvate kinase type M2 (PKM2) upregulation [88]. PKM2, a major protein in glycolysis, is known to be upregulated in many cancer types. In non-small cell lung cancer (NSCLC), hypoxic TDEVs contain PKM2. Upon delivery to NSCLC cells, PKM2 promoted glycolysis to produce reductive metabolites, which neutralized cisplatin-induced ROS, thus conferring chemotherapy resistance. Additionally, PKM2 inhibited apoptosis in a PKM2-BCL2-dependent manner. Furthermore, EV PKM2 EVs reprogrammed CAFs to create an acidic microenvironment, promoting NSCLC cell proliferation and cisplatin resistance [89]. Interestingly, one PKM2 study revealed a non-metabolic activity, in which PKM2 controls the release of EVs [90]. This suggests a positive feedback loop wherein TDEVs cause PKM2 upregulation in cancer and CAF cells, which in turn increases the release of TDEVs and treatment resistance.

The ability of the protein cargo of EVs to contribute to cancer progression and resistance has also been described in ovarian cancer. EVs from hypoxic ovarian cancer cells are enriched with signals associated with metastasis and glycolysis. Moreover, proteins associated with the glycolytic proteome found in those EVs do not reflect the cell of origin, thus suggesting the specific packaging of proteins into EVs. These EVs released from hypoxic cells confer chemotherapeutic resistance to recipient normoxic cancer cells [91]. Analysis of plasma-circulating EVs from ovarian cancer patients who suffered from recurrence identified four exosome-associated glycolytic pathway proteins: PKM1/2, enolase 1, glyceraldehyde-3-phosphate dehydrogenase, and aldolase fructose-bisphosphate. ROC curve analysis of the expression levels of these proteins efficiently identified the risk of ovarian cancer recurrence [91].

One of the most important chemotherapy-resistant mechanisms known to researchers is the overexpression of ATP-binding cassette (ABC) transporters, commonly known as drug efflux pumps, such as P-glycoprotein (P-gp) [92], which plays a significant role in multidrug resistance (MDR). P-gp transports drug substrates across the cell membrane, thus decreasing their intracellular concentrations. Shen et al. have shown that EVs derived from chemotherapy-treated breast cancer cells induced a cancer stem-like cell phenotype and conferred cancer cells with resistance to therapy, which arose from the downregulation of the transcription factor One Cut homeobox 2 (ONECUT2) and an increase in ABC transporters [93]. Lopes-Rodrigues et al. investigated the difference between MDR cell lines (chronic myeloid leukemia and non-small cell lung cancer) and their drug-sensitive matching pairs. They revealed that the greatest difference between MDR cells and their drug-sensitive counterparts occurred in metabolic processes, specifically glycolysis, the pentose phosphate pathway, and glutathione metabolism. EVs from MDR cells were able to transfer their metabolic phenotype to the sensitive cells [94].

Radiation therapy (RT) is the most widely used therapeutic method for cancer treatment. RT-resistant tumor cells survive and lead to local recurrence and distant metastasis, which is responsible for treatment failures and mortality. One of the results of radiation therapy is the generation of ROS. EVs have been shown to affect both the generation of ROS, as well as protection from ROS damage [95]. EVs from irradiated cancer cells confer radiotherapy resistance via induction of the DNA repair mechanism [96] and by transferring migratory phenotype-supporting proteins to recipient cancer cells [97]. On the other hand, mesenchymal stem cell-derived EVs can also enhance RT-induced cell death in tumors [98]. Evidence for metabolic reprogramming in RT was presented by Wang et al., who investigated the effect of irradiated lung cancer cell EVs on non-irradiated cancer cells. They described the transfer of metabolic enzymes, ALDOA and ALDH3A1, which facilitated glycolytic activity in recipient cancer cells. This glycolytic enhancement directly affected lung cancer cell motility and metastasis [99]. In NPC, EV-packaged, LMP1-activated CAFs promoted radiation resistance of tumor cells, although the exact mechanism related to this remains unknown [48].

### 2.4. Angiogenesis

Another process required for tumor development and propagation is the creation of new blood vessels to supply oxygen, metabolites, and an effective way to remove waste products [100]. EVs have been shown to encourage angiogenesis in different mechanisms [101,102]. Nevertheless, the metabolic reprogramming of EVs that foster angiogenesis requires more extensive investigation. EVs derived from surgically resected, viable CRC tissues carry one of the cationic amino acid transporter family proteins, high-affinity cationic amino acid transporter 1 (CAT1), which is considered to be the major carrier of arginine, lysine, and ornithine. CAT1-overexpressed EVs have drastically enhanced vascular endothelial cell growth and tubule formation via the upregulation of arginine transport and arginine-oriented metabolic and phosphorylation pathways [103].

HNSCC produced EVs that carry on their surface adenosine and the ectonucleotidases CD39 and CD73, which both enzymatically produce adenosine. These EVs interact with endothelial cells, inducing an A2BR-mediated stimulation of endothelial cell growth. Moreover, adenosine from TDEVs binds to A2BR on macrophages and stimulates the secretion of proangiogenic factors such as angiopoietin-1, endothelin-1, platelet factor 4, and serpin E1, all of which stimulate angiogenesis [104]. AML-derived EVs contain vascular endothelial growth factor (VEGF), VEGF receptor (VEGFR) messenger RNA, and induce VEGFR expression in endothelial cells. These EVs enhance glycolysis, as well as the proliferation of endothelial cells, which confer chemoresistance to AML cells [105].

### 2.5. Cachexia and Other Systemic Effects

Muscle wasting, typical in cancer patients, is one of the main features characterizing cancer cachexia, a complex syndrome associated with reduced survival, poor quality of life, and decreased tolerance to anticancer treatments. Cell-line-derived EVs from Lewis lung carcinoma and colon carcinoma have been found to significantly affect energy metabolism in myotube cultures by reducing mitochondrial respiration and increasing lactate production. They also impaired the differentiation of murine myoblasts in vitro. Moreover, EVs isolated from the plasma of tumor-bearing animals partially recapitulated cachexia when infused into healthy animals [106].

PC is one of the deadliest tumor types, which sees most patients dying within 6 months. A major contributor to early mortality is the profound and rapid loss of adipose tissue (AT) and skeletal muscle mass. This weight loss is paradoxically associated with the development of new-onset diabetes. Sagar et al. have shown that EVs from patient-derived PC cell lines transfer adrenomedullin (AM) to adipocytes, which promotes lipolysis. AM, a PC-secreted, pluripotent hormone has been identified as inducing β-cell dysfunction and decreased insulin secretion, and is therefore associated with PC-induced diabetes [107]. Another assumed mechanism for new-onset diabetes involves the induction of insulin resistance. In this regard, Wang et al. have shown that PC-derived exosomes (but not microvesicles) trigger an eventual state of insulin resistance in skeletal muscle cells at least partially through the inhibition of PI3K/Akt signaling, thus impairing Glut4 trafficking to the plasma membrane [108].

## 3. Tumor Microenvironment-Derived EVs

**Figure 2:**
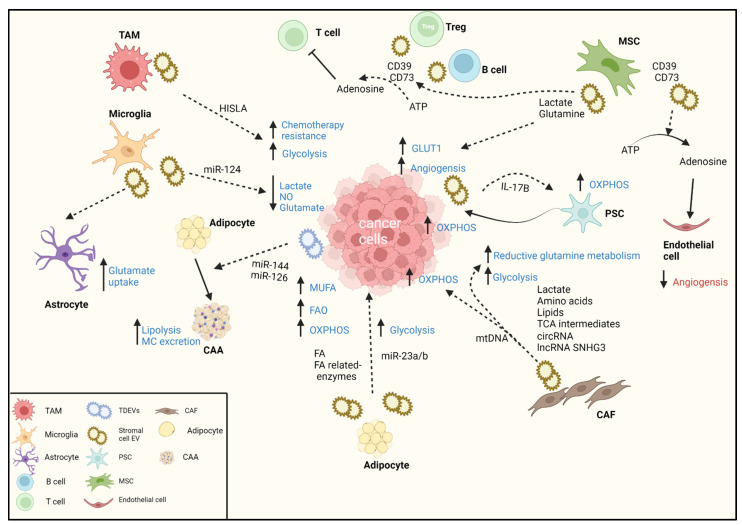
Tumor microenvironment metabolic reprogramming by stromal-derived extracellular vesicles. There is crosstalk between stromal cells and cancer cells. The metabolic landscape is dynamic and depends upon the availability of nutrients and complex TME communication. This can result in opposing effects for EVs. Stromal EVs transfer different metabolites to support cancer cells. CAFs transfer amino acids and TCA cycle intermediates to cancer cells to induce glycolysis and reductive glutamine metabolism. On the other hand, CAF EVs also enhance the cancer cell OXPHOS via the transfer of mtDNA. Immune cells transfer miRNA and HISLA to augment glycolysis. MSC EVs have dual effects—supplying metabolites (lactate and glutamine) to replenish cancer cells and support angiogenesis, as well as hydrolyzing ATP to adenosine, which can inhibit angiogenesis. Adenosine hydrolyzation by B and Treg cells binds to T cells, prompting immunoevasion. Adipocytes shed EVs that can increase glycolysis but can also increase OXPHOS and FAO levels without changing that activity. TAM—tumor-associated macrophage; CAF—cancer-associated fibroblast; TME—tumor microenvironment; OXPHOS—oxidative phosphorylation; MSC—mesenchymal stem cell; PSC—pancreatic stellate cell; FA—fatty acid; FAO—fatty acid oxidation; NO—nitric oxide; TCA—tricarboxylic acid; HISLA—HIF-1α-stabilizing long noncoding RNA MC—monocarboxylic acid; CAA—cancer-associated adipocytes. Dashed arrow—transferred EV cargo.

TME cells are not only affected by cancer cells; they also reciprocally influence cancer cell metabolism. This section offers a review of the evidence supporting reprogramming of the metabolism of cancer cells and stromal cells by stromal-derived EVs (Figure 2).

### 3.1. Immune Cells

A major portion of any tumor mass is composed of immune cells, which have a tremendous effect on the behavior of tumors—whether by constraining them or supporting their progression.

Macrophages—a heterogeneous population of cells with the ability to differentiate depending on circumstance—also occupy a significant portion of tumors, and the relation between the macrophage burden of a given tumor to prognosis was established in 1970 [109]. Classically-activated macrophages are known as *M1 killers* because their activation is related to TH1 cytokines and directed toward inflammatory, anti-bacterial responses. Similarly, alternatively-activated macrophages are known as *M2 repair macrophages* due to their association with TH2 cytokines and their role in wound healing. Tumor-associated macrophages (TAMs) are a well-described subpopulation of macrophages, which bear a resemblance to M2 macrophages, as they contain high levels of M2 markers and low levels of M1 markers. TAMs are the most abundant immune cells within the TME and are associated with poor patient outcomes in multiple types of cancer [110].

Several studies have linked macrophage polarization to cancer-derived EVs. Most, but not all, of the evidence suggests that TDEV polarization tends towards the M2 phenotype. This has been shown in CRC, OCSCC, ovarian, hepatocellular carcinoma, glioblastoma, breast, prostate, and, pancreatic cancers. Furthermore, a mutual relationship between TAM EVs and breast cancer has also been described, which shows that the former enhances aerobic glycolysis and apoptotic resistance of the latter’s cells via the transmission of HIF-1α-stabilizing long noncoding RNA (HISLA). In return, glycolytic tumor cells release lactate, further upregulating HISLA in macrophages, thus constituting a positive feedback loop between TAMs and tumor cells. Blocking EV-transmitted HISLA inhibits glycolysis and chemoresistance of breast cancer in vivo. Clinically, HISLA expression in TAMs is associated with enhanced glycolysis, poor chemotherapeutic response, and decreased survival of patients with breast cancer [111].

Similar to peripheral macrophages, the primary innate immune effector cells of the CNS are microglia. Derived from these are small EVs that transfer miR-124 to glioma cancer cells and modify glioma cell metabolism by reducing the release of lactate, nitric oxide, and glutamate. Moreover, EVs also affect glutamate homeostasis, increasing the expression of glutamate transporter (Glt-1) on astrocytes. In glioma-bearing mice, the in vivo benefits of small, microglia-derived EVs are a significantly reduced tumor mass and increased survival, which is mediated by miR-124 [112]. Cianciaruso et al. investigated the proteomics and lipidomics of TAM EVs and revealed that their enrichment for bioactive lipids and biosynthetic enzymes may alter pro-inflammatory signaling in cancer cells [113].

As described in the immunoescape section above, as adenosine inhibits the T cell response, it is a major immunosuppressive metabolite. In keeping with this, since adenosine can be produced by immune cell EVs, B-cell-derived EVs have been shown to express high levels of CD39 and CD73. These hydrolyze ATP, which is released by chemotherapy-treated tumor cells, into adenosine and attenuate chemotherapeutic efficacy by constraining CD8+ T cell responses in melanoma, as well as colon and breast cancer models in vitro and in vivo. Moreover, tumor B cells have increased the production of EVs through HIF-1α, enhancing Rab27a transcription in tumor B cells [114]. This suggests positive feedback, whereby tumor cells produce lactic acid (via enhanced glycolysis), which stabilizes HIF-1α [115], in turn increasing tumor B cell EVs, which results in immunosuppression. Similarly, CD73 has been found on Treg cell-derived EVs. Accordingly, when these exosomes were incubated in the presence of adenosine-5-monophosphate, adenosine production was observed. Positive CD73 Treg-derived exosomes (but *not* negative CD73 Treg-derived exosomes) suppressed CD4+CD25− T-cell proliferation [116].

### 3.2. Cancer-Associated Fibroblasts

The impact of Cancer-Associated Fibroblast (CAF)-derived EVs has also been described as being partially responsible for tumor cell survival in the hostile, nutrient-deprived, and hypoxic environment of PC. CAF-derived EVs from these patients have been found to contain several metabolites, including lactate, acetate, amino acids, lipids, and tricarboxylic acid (TCA) cycle intermediates. Additionally, CAF EVs derived from PC tumor cell macropinocytosis have been found to block OXPHOS by inhibiting the electron transport chain. Provided in its place were a plethora of intermediate metabolites, lipids, and amino acids, which thus allowed cancer cells to favor glycolysis, reductive glutamine metabolism, and enhanced proliferation [117]. Similarly, a 2020 study showed that CAFs transfer circRNA via EVs to hepatocellular carcinoma (HCC), thereby enhancing glycolysis through regulating HK2 expression. Enhanced glycolysis translated into increased viability and invasion both in vitro and in vivo [118]. In the same manner, CAF-secreted EVs were shown to transfer lncRNA SNHG3, which served as a molecular sponge for miR-330-5p in breast cancer cells. PKM2 is a direct target of miR-330-5p; therefore, CAF transfer of SNHG3 to breast cancer cells decreases miR-330-5p levels and increases PKM2 expression, inhibits mitochondrial OXPHOS, increases glycolysis carboxylation, and enhances breast tumor cell proliferation. Correspondingly, SNHG3 knockdown in CAF-secreted exosomes suppressed glycolytic metabolism and cell proliferation in tumor cells [119]. 

Although the glycolytic pathway is considered to be one of the most altered metabolic pathways in cancer, such an alteration does not always occur, as the effects of TME-derived EVs are sometimes contradictory. For example, in contrast to the aforementioned enhanced glycolysis and suppressed OXPHOS, hormonal therapy-resistant (HTR) breast cancer has been shown to have increased OXPHOS in comparison to hormonal therapy-sensitive (HTS) cancer cells. Furthermore, the progression from HTS to dormant cells, and eventually to HTR has been attributed to an increase in OXPHOS. Sansone et al. found that CAF-derived EVs contain full mitochondrial DNA, which they transfer to HTS and dormant cancer cells, transforming them into HTR and promoting their growth by increasing the OXPHOS pathway. This suggests that breast cancer regulates its metabolism through the acquisition or removal of mtDNA from TME EVs [120].

### 3.3. Mesenchymal Stem Cells

Mesenchymal stem cells (MSCs) are multipotent cells that reside in the majority of human tissues and organs. Each organ contains a specific population of stem cells, which maintains the regenerative process for the tissue where they reside, although some of these have greater plasticity and can differentiate into multiple cell lineages [121]. Studies on the metabolic effects of MSC-derived EVs are scarce and offer only indirect evidence of this, let alone the fact that yielded results have been inconclusive. Human bone marrow MSC-derived EVs promoted osteosarcoma growth via upregulation of PI3K/AKT and HIF-1α, as well as downstream proteins such as GLUT1, which is the main transporter of glucose into the cell [122]. Likewise, human MSC-derived EVs have been shown to carry a complex cargo, including lactic acid and glutamine, which supported breast cancer proliferation and angiogenesis [123]. It is important to note, however, that the authors did not confirm that tumor proliferation was due to the metabolic cargo, as opposed to other elements of the EV load (e.g., miRNA, proteins, etc.).

In a different study, MSCs were cultured in a hypoxic and nutrient-poor environment to emulate the TME, and their corresponding EVs were subject to metabolic analysis. EVs from hypoxic MSCs contained 21 distinct metabolites that have been directly associated with immunoregulation—specifically M2 macrophage polarization and regulatory T lymphocyte induction—thus suggesting a role for MSC EVs in immunoevasion [124]. On the other hand, EVs from AT-derived MSCs were internalized into ovarian cancer cell lines, which subsequently showed decreased metabolic activity that translated into decreased proliferation [125]. In response to pro-inflammatory cytokines, bone marrow MSCs release EVs enriched in CD39 and CD73, which hydrolyze extracellular ATP to produce adenosine. The binding of adenosine to A_2B_AR inhibited in vitro migration of endothelial cells. In vivo, MSC-EVs inhibited angiogenesis in breast cancer and fibrosarcoma models [126].

### 3.4. Pancreatic Stellate Cells

Pancreatic stellate cells (PSCs) constitute a major stromal component and form a niche for cancer stem cells. In healthy pancreatic tissue, PSCs are quiescent. EVs from PC carry IL-17B, which activates stromal PSCs and induces the expression of IL-17BR. Consequently, PSCs increase OXPHOS while reducing mitochondrial turnover. PSCs activate tumor cells in a feedback loop. Tumor cells subsequently increase OXPHOS and decrease glycolysis via IL-6. In a co-injection xenograft mouse model, IL-17BR overexpression in PSCs accelerated tumor growth in vivo [127].

### 3.5. Adipocytes

Excess weight and obesity are associated with an increased risk for at least 13 types of cancer. It is estimated that 40% of cancer incidence is attributable to obesity- and overweight-related cancers [128]. Correspondingly, weight loss is associated with a reduced risk for all cancers [129]. Adipocytes, the main cellular components of AT, have been shown to promote tumor progression [130]. Classically, studies on the link between adipocytes and cancer concentrated on soluble factors, and recently the role of adipocyte EVs has been found in cancer. Adipocyte-derived EVs transfer proteins involved in lipid metabolism, specifically fatty acid oxidation (FAO) to cancer cells. The transfer of FAO-related proteins stimulated FAO in tumor cells and contributed to an increase in mitochondria numbers and density without altering glycolytic activity. This metabolic shift resulted in enhanced migration and invasion of melanoma, as well as prostate cancer cells [131]. Later, it was shown that adipocyte EVs stimulate melanoma FAO by providing both enzymes and substrates. In fact, in obesity, the effect of EVs depends on the transport of fatty acids, not on the transfer of FAO-related enzymes [132].

HCC patients with a high body fat ratio (BFR) were shown to have upregulated levels of miR-23a/b in both serum EVs and tumor tissue, in comparison to patients with low BFRs. In vitro studies have suggested that miR-23a/b was most likely to have been derived from adipocytes and transported into cancer cells via EVs. Overexpression of miR-23a/b reduced the expression level of von Hippel–Lindau (VHL) protein and upregulated its downstream targets, including HIF-1α, GLUT-1, and VEGF. In vitro and In vivo studies confirmed the effects of the miR 23a/b-VHL-HIF-1α axis on tumor growth and chemoresistance [133]. In NPC, hypoxic adipocyte-derived EVs reduced the expression of miR-433-3p, resulting in increased levels of stearoyl-CoA desaturase 1 (SCD1), which is the key regulatory gene for the synthesis of monounsaturated fatty acids (MUFAs) and plays an important role in the lipid metabolism of tumors [134]. The increased expression of SCD1 promoted proliferation, migration, and lipid accumulation by NPC cells [134].

TDEVs have also been shown to affect and harness adipocytes for tumor progression. For example, breast cancer-derived EVs shuttled to resident adipocytes resulted in their conversion to cancer-associated adipocytes (CAAs). TDEVs induced beige/brown differentiation and reprogrammed metabolism in stromal adipocytes, which in turn altered tumor metabolism, specifically the accumulation of triglyceride and an increase in glucose uptake. This altered metabolism promoted tumor cell invasiveness in vitro [135].

## 4. Crosstalk between Tumor EVs and Stromal EVs

The transfer of EVs has a bidirectional effect on the communication between cancer and stromal cells. For example, CAFs secrete EVs that provide metabolites and inhibit OXPHOS in PC. Consequently, glutamine undergoes reductive carboxylation to replenish TCA intermediates [117]. This converts glutamine to glutamate and ammonia. Ammonia diffuses out of cancer cells and then promotes an autophagic microenvironment, which in turn supplies more metabolites to cancer cells [118]. Another example is the transfer of EV-HISLA from TAMs to breast cancer cells, which increases aerobic glycolysis and lactate production. Lactate released from cancer cells further upregulates HISLA in TAMs, which creates a feed-forward loop between TAMs and cancer cells [111].

There are probably more complex interactions that encompass several cell types in the TME. For example, TDEVs polarize macrophages toward the TAM phenotype through multiple reported mechanisms [136,137,138]. As explained above, TAMs secrete HISLA-containing EVs that are shuttled into the TME to cancer and stromal cells, including B cells. HISLA stabilizes HIF1α, which increases EV production and elevates the release of EVs by B cells [114]. In turn, B cell EVs attenuate postchemotherapy CD8 T cell responses. This demonstrates the complexity and therapeutic potential of targeting EVs in the TME.

## 5. Discussion

A tumor is much more than the collectivity of its cancer cells. TME targeting could bolster conventional treatments and improve therapeutic outcomes. The TME is a complex ecosystem consisting of cells and an extracellular matrix, each of which affects tumorigenesis differently. Cancer cells need large amounts of ATP and macromolecules to sustain their proliferation, which is not easily accomplished within the poor environment of tumors themselves. However, one of the hallmarks of cancer is its ability to alter its metabolism precisely to sustain proliferation and survival inside a harsh environment that is both oxygen- and nutrient-deprived. EVs are major carriers of information within the TME. They convey proteins, amino acids, lipids, and nucleic acids that alter the function of recipient cells. In this review, we have presented evidence for TME EV involvement in the metabolic reprogramming of cancer and stromal cells. Most of the evidence presented supports the notion that EVs favor cancer progression by enhancing angiogenesis, proliferation, metastasis, treatment resistance, and immunoevasion.

Other areas of research not reviewed here include the use of EVs in cancer diagnostics and prognostics. Within the diagnostic field (research, pharmaceutical development, and healthcare industries), there is growing interest in EVs, as they possess several advantages over traditional screening methods. Included here is the fact that they are released by all cells and in high quantities (especially in comparison to circulating tumor cells or circulating tumor DNA). Additionally, they also remain stable under different conditions and within a variety of bodily fluids, which thus protects their contained cargoes (and possible biomarkers). This stability also facilitates cargo detection in subsequent analyses. For example, an analysis of plasma-circulating EVs from ovarian cancer patients who suffered from recurrence identified four EV-associated glycolytic pathway proteins, the expression levels of which efficiently identified the risk of further recurrence [91]. For detailed reviews of EV use in cancer diagnostics, see [139,140,141].

Another growing body of EV research focuses on their use as a therapeutic tool. Reducing the metabolic effects of EVs by suppressing their biogenesis or release is a valid objective that continues to be investigated. A more attractive option is the development of EVs as drug delivery vehicles. Here, too, EV stability is a benefit, but there are additional features that make them appealing for this purpose. For instance, some studies have shown that EVs display inherent targeting properties that are dictated by their lipid composition and protein content [142].

More importantly, EVs and their parent cells can be manipulated to improve their capability to deliver drugs to recipient cells. Manipulated EVs can also have longer circulation times, protection from unwanted phagocytosis, and better selectivity profiles [143]. An example of manipulating EVs to target TME metabolism was offered by Li et al., who noted that CAF-secreted EVs carry SNHG3, which enhances glycolysis, downregulates OXPHOS, and promotes proliferation. These effects were reversed by the knockdown of SNHG3 in CAFs, which was mediated by PKM downregulation. Breast cancer patient-derived CAFs were transduced with sh-SNHG3-expressing lentivirus to obtain stable SNHG3 knockdown cells. In vivo, the knockdown cells (as compared to SH-control transduced cells) shuttled EVs lacking SNHG3, thereby decreasing PKM expression, which resulted in inhibited tumor proliferation, heightened intratumoral pH, and decreased lactate levels [119].

## 6. Conclusions

EV crosstalk between cancer and stromal cells reprograms the localized metabolic activity of the TME itself (i.e., cancer and stromal cells), as well as the metabolic activity of the distant TME (i.e., the premetastatic niche, muscles, and adipocytes), thus favoring cancer progression. Targeting the communication mechanisms and systems utilized by EVs therefore represents a new frontier in the expansion of the tools available to medical science in the fight against cancer.
